# Draft Genome Sequence of Dyella sp. Strain C11, Isolated from a Malaysian Tropical Peat Swamp Forest

**DOI:** 10.1128/genomeA.00459-18

**Published:** 2018-06-21

**Authors:** Chin Chin Too, Kuan Shion Ong, Sui Mae Lee, Catherine M. Yule, Alexander Keller

**Affiliations:** a School of Science, Monash University Malaysia, Subang Jaya, Selangor, Malaysia; b Tropical Medicine & Biology Multidisciplinary Platform, Monash University Malaysia, Subang Jaya, Selangor, Malaysia; c Department of Bioinformatics, Biocenter, University of Würzburg, Würzburg, Germany; d Center for Computational and Theoretical Biology, University of Würzburg, Würzburg, Germany

## Abstract

We report here the draft genome sequences of a bacterial isolate, Dyella sp. strain C11, which was isolated from a Malaysian tropical peat swamp forest. The putative genes for the biogeochemical processes were annotated, and the genome was deposited in an online database.

## GENOME ANNOUNCEMENT

Tropical peat swamp forests (TPSF) are extreme ecosystems best known for their acidic, anoxic, and nutrient-depleted conditions (1). They serve as one of the largest terrestrial carbon reservoirs and thus play a role in regulating global climate. Dyella sp. strain C11 was isolated from the surface peat sampled from the North Selangor Peat Swamp Forest in Malaysia, and it was sequenced to investigate putative functional genes involved in carbon and nitrogen cycles. Dyella spp. are Gram-negative bacteria, and several species have been isolated from soil (2–4).

Bacterial culture and isolation were conducted at Monash University Malaysia. The surface peat sample was first enriched in primary enrichment medium (5), followed by bacterial cultivation on minimal medium using methanol as the carbon source (6). DNA extraction was performed according to the protocol of the QIAamp DNA Mini Kit (Qiagen, Hilden, Germany), using the pure culture incubated in tryptic soy broth for 36 h at 30°C. Library preparation was conducted based on the protocol of the Nextera XT DNA library preparation kit (Illumina, San Diego, CA), followed by whole-genome sequencing on the Illumina NextSeq 500 platform (150-bp paired-end reads) at the Biocenter, University of Würzburg, Germany. DNA raw reads were corrected and assembled *de novo* using SPAdes version 3.10.1 (7), followed by gene annotation in Prokka version 1.12 (8) and BlastKOALA (9). The Enzyme Commission (EC) numbers from the output of Prokka and the KEGG orthologs (KO) assigned by BlastKOALA were used to map the metabolic pathways on the Kyoto Encyclopedia of Genes and Genomes (KEGG) database (10).

There were in total 46 contigs observed, with a total length of 4,730,309 bp (*N*_50_, 174,168 bp) and 63.28% GC content. There were 4,277 coding DNA sequences (CDSs), 1 transfer-messenger RNA (tmRNA), 3 rRNAs, and 51 tRNAs found within the genome. Dyella sp. strain C11 was predicted to encode various hydrolases, such as α-amylase, cellulase, endo-1,4-β-xylanase, oligo-1,6-glucosidase, chitinase, β-glucosidase, β-glucuronidase, and xylan α-1,2-glucuronosidase, which catalyze the hydrolysis of carbohydrates and plant cell wall materials. Regarding nitrogen metabolism, the nitric oxide reductase subunit B gene (*norB*) was observed, indicating its potential role in converting nitric oxide to nitrous oxide, which is a greenhouse gas (11).

### Accession number(s).

This whole-genome shotgun project has been deposited at DDBJ/ENA/GenBank under the accession number QAVX00000000. The version described in this paper is version QAVX01000000.
